# Critical Flicker Fusion Frequency: A Narrative Review

**DOI:** 10.3390/medicina57101096

**Published:** 2021-10-13

**Authors:** Natalia D. Mankowska, Anna B. Marcinkowska, Monika Waskow, Rita I. Sharma, Jacek Kot, Pawel J. Winklewski

**Affiliations:** 1 Applied Cognitive Neuroscience Lab, Department of Human Physiology, Medical University of Gdansk, 80-210 Gdansk, Poland; anna.marcinkowska@gumed.edu.pl; 2 2nd Department of Radiology, Medical University of Gdansk, 80-210 Gdansk, Poland; pawel.winklewski@gumed.edu.pl; 3 Institute of Health Sciences, Pomeranian University in Slupsk, 76-200 Slupsk, Poland; monika.waskow@apsl.edu.pl; 4 Department of Human Physiology, Medical University of Gdansk, 80-210 Gdansk, Poland; rita.sharma@gumed.edu.pl; 5 Department of Anaesthesiology and Intensive Care, Medical University of Gdansk, 80-210 Gdansk, Poland; 6 National Centre for Hyperbaric Medicine, Institute of Maritime and Tropical Medicine in Gdynia, Medical University of Gdansk, 80-210 Gdansk, Poland; jkot@gumed.edu.pl

**Keywords:** critical flicker fusion frequency, threshold of flicker fusion, neuropsychology, diving and hyperbaric medicine, minimal hepatic encephalopathy

## Abstract

This review presents the current knowledge of the usage of critical flicker fusion frequency (CFF) in human and animal model studies. CFF has a wide application in different fields, especially as an indicator of cortical arousal and visual processing. In medicine, CFF may be helpful for diagnostic purposes, for example in epilepsy or minimal hepatic encephalopathy. Given the environmental studies and a limited number of other methods, it is applicable in diving and hyperbaric medicine. Current research also shows the relationship between CFF and other electrophysiological methods, such as electroencephalography. The human eye can detect flicker at 50–90 Hz but reports are showing the possibility to distinguish between steady and modulated light up to 500 Hz. Future research with the use of CFF is needed to better understand its utility and application.

## 1. Introduction

Critical flicker fusion frequency (CFF or CFFF) is defined as the frequency at which flickering light can be perceived as continuous and it is used to assess the processing of temporal vision. The upper level of one’s abilities in visual processing is described as the critical flicker fusion threshold (or threshold for flicker fusion, TFF), which represents the maximum speed of flickering light that can be perceived by the visual system [1,2]. Because of its efficiency in detecting rapid changes, it is used as an index of cerebral nervous system (CNS) function that is described as alertness and cortical arousal in humans [3,4].

The ability to detect flicker fusion is dependent on: (1) frequency of the modulation, (2) the amplitude of the modulation, (3) the average illumination intensity, (4) the position on the retina at which the stimulus occurs, (5) the wavelength or colour of the LED, (6) the intensity of ambient light [3,5,6] or (7) the viewing distance and (8) size of the stimulus [7]. Moreover, there are also internal factors of individuals that can affect CFF measures: age, sex, personality traits, fatigue, circadian variation in brain activity [4] and cognitive functions like visual integration, visuomotor skills and decision-making processes [3]. The performance of CFF in humans and predators alike is dependent on these factors. Umeton et al. also describe preys’ features like a pattern or even the way they move as relevant in perceiving the flicker fusion effect [2].

It is believed that the human eye cannot detect flicker above 50 to 90 Hz and it depends on intensity and contrast, but some reports indicate people can distinguish between modulated and steady light at up to 500 Hz [8].

In recent years, there have been many studies with animal models. The pioneers of these studies were Shure and Halstead who investigated the influence of brain lesions in monkeys on TFF [9]. The reason for this decision was Halstead’s finding that the removal of the specific brain localisation relates to CFF performance. Nowadays, not only monkeys are of interest to scientists. Lisney and colleagues used electroretinograms and indicated that flicker from fluorescent lamps may be a stress factor for hens [10,11]. The limitation of these studies is the number of included chickens (4 to 15), thus future research is needed.

A high CFF threshold is crucial for flying animals (like pigeons—143 Hz or peregrine falcons—129 Hz) that need an efficient visual system, e.g., to detect rapidly approaching objects to avoid colliding with them, but there are some studies with shrimps that need similar skills. The shrimp TFF is about 160 Hz, although there are individuals with a threshold of 200 Hz [12]. These are just a few examples of the use of CFF in animal studies; in the literature, reports may be found on its usage in dogs, mice, rats or snakes and more. Researchers typically use two types of method to determine the CFF of an animal—electroretinograms and behavioural methods, like two-alternative forced-choice procedure [13,14], e.g., pecking at the lit panel by a bird [15].

Flicker has its countertype in a different sensory modality, i.e., hearing. A few researchers have done a comparison of visual and auditory stimuli. Shipley found that in healthy and young individuals, critical flicker frequency is worse than a critical flutter frequency (which is “the frequency at which a clicking sound appears steady”) [16]. Shams et al. point out that the perception of visual stimulus intensity may be modulated by the occurrence of sound [5]. Moreover, the frequency of flickering light is prone to change under the influence of the frequency of fluttering sound; multiple audio signals with a single flash result in perceiving this as multiple flashes.

The CFF test may be used in different forms, but the instruction is usually the same; a subject has to focus their vision on a light-emitting diode when the light frequency is increased at a constant rate (e.g., 1 Hz) and has to press a button when it seems to be continuous light in their opinion. It is also possible to reverse the order of the task to one in which the light frequency decreases and the subject has to report when they see flicker.

In some studies, the CFF test is used as a computer program where participants have to report altered vision by pressing a button [17]; in others, it is a device that can be described as similar to virtual reality glasses, for example, the HEPAtonorm Analyzer [18] or with the addition of other methods like electroretinograms or functional magnetic image resonance, among others [10]. The choice of (1) the device to measure TFF and (2) additional methods should depend on the research hypotheses and the research group.

## 2. Arousal as an Indicator of Cognitive Performance

The relationship between arousal and cognitive performance has been known since 1908, when two psychologists, Robert M. Yerkes and John D. Dodson, did a series of experiments with mice and described the Yerkes-Dodson Law, which states that cognitive performance increases with both physiological and mental arousal, but when it becomes too high, the performance decreases. This law could be illustrated in a bell-shaped curve [19]. The critical flicker fusion frequency test can be used as a tool to monitor these changes in brain function and to assess cortical arousal in various environmental conditions [3,20,21]. Additionally, arousal can be modulated by physical activity. Based on this knowledge, several experiments investigated the impact of physical exercise on the CFF performance and found that arousal increases directly after exercise and returns to the output level during recovery [21].

Tomporowski et al. compared results from the Paced Auditory Serial Addition Test (PASAT) before and after exercise. In Experiment 1, nine men completed two sessions of a 40-min bout of cycling, and in Experiment 2, 10 women completed four 120-min sessions of cycling [22]. Researchers have proven that arousal is exercise-induced and may induce better PASAT performance, which means that acute aerobic exercise may affect working memory and attention.

Lambourne and colleagues assessed the influence of aerobic exercises (on cycle ergometer) on two mental processes: sensory discrimination and executive functions in 19 young adults [21]. They used the CFF test as a visual sensory-discrimination task. The measures of the CFF performance were made five times during 40 min of cycling at a moderate level and three times during the 30-min post-exercise period. The results showed an improvement in cognitive performance after physical exercise. However, the CFF results rapidly decreased after the termination of exercise. This means that the increasing level of CFF may result from enhancing receptiveness to sensory stimulation involved in stimulus detection. The arousal probably does not influence executive processing, but the effect of acute exercise on higher-level processes is unclear.

## 3. Use of the Critical Flicker Fusion Test in Neuropsychology

The use of CFF for CNS function assessment is postulated by Casey et al. [23]. In experimental procedures with electroretinogram and functional magnetic resonance imaging researchers found that subjects report fusion much longer after the retina and visual cortex responded to flicker [24,25] which means that the human perception of flicker is associated with the activity of higher cortical regions [26,27].

The Halstead-Reitan Battery of Neuropsychological Tests was initially used as a method to assess cognitive functioning in individuals with brain lesions above 15 years old. It was created in 1947 [28] and included two critical flicker fusion measures that were dropped later because of insufficient discrimination between groups of patients with or without brain lesions [29].

CFF as a method to assess cognitive functions is objective, simple, quick, low-cost and it is not affected by factors such as a level of education or language [30,31,32,33]. A significant advantage of CFF performance is its resistance to the learning effect [34,35,36].

Studies with CFF can be performed in 3-month-old infants [37]. They estimate infant TFF by a two-alternative forced-choice preferential looking technique. The researcher (observer) estimates the location of the flickering target (left or right side of the visual field) based on cues given by the infant (e.g., gaze direction or length of looking time to each side). If 75% or more trials were well estimated, then it was inferred that the infant was able to detect flicker at that frequency. The location of light was semi-randomised, and the first trials began at 20 Hz. The findings indicate a significant improvement in development is occurring between 3 to 4.5 months of age, which then may slow or even plateau. The forced-choice preferential looking procedure is not the only method used in research involving infants; researchers also use visual evoked potentials and electroretinograms [38].

The differences in CFF performance (as an index of processing speed) in children might be a predictor of future cognitive functioning. Visual processing speed, however, is not the only predictor of cognitive abilities in the future [1]. Saint and colleagues examined 54 children with CFF and the Woodcock-Johnson III Tests of Cognitive Abilities and observed that psychomotor coordination became better with age (younger children had longer reaction time latency) [1].

The CFF test in children or adolescents has also been used in studies with groups with brain injuries [18], diabetes [39] and reading disabilities [40].

## 4. The Diagnostic Values of CFF

Several studies have investigated the effect of dietary supplementation, especially with compounds that are naturally found throughout the CNS (e.g., lutein, fatty acids or plant pigments, xanthophylls, found in the brain and retina in particular), with the threshold of CFF [41,42,43]. Bovier et al. have indicated improvements in reaction times and increasing CFF thresholds in adults after lutein and zeaxanthin supplementation [41,42]. Other research projects support this hypothesis [44,45] and even use CFF to find drug effects on psychomotor performance, attention and concentration [46].

Lauridsen et al. compared a continuous reaction times test (CRT) with CFF for the diagnosis of minimal hepatic encephalopathy [47]. The CRT test was a measure of the subject’s ability to perform motor reactions adequately and repeatedly. The CFF test reflected biological activity in retinal cells and provided information about visual processing, arousal and attention. The patient’s CFF threshold was the average of the nine measurements at 60 Hz, and if it was lower than 39 Hz, then it was considered cerebral dysfunction. In the trial with CRT, participants were asked to press the button as soon as they heard the signal at 500 Hz and 90 dB. A reaction time above 2 s was registered as a lack of response. Both CRT and CFF tests gave false positives and inconsistent results. These two measures describe different aspects of minimal hepatic encephalopathy so the choice between the CRT or CFF test should be made carefully.

The CFF threshold may be a useful measure to follow cognitive processing abilities in patients with implanted vagus nerve stimulators (VNSs) for epilepsy treatment and Alzheimer’s disease [17,48]. After a 12-month treatment with a VNS, all epilepsy patients showed significant improvement in CFF compared to baseline (*p* < 0.05).

Some research has suggested that a reduced CFF threshold could be used to detect individuals with Alzheimer’s disease [27,48,49,50,51]. In these patients, impairment of short-term memory is observed, which could be enhanced by the 10 Hz flicker. This result confirms previous ones that found memory dysfunction correlated with the loss of the 10 Hz alpha rhythm [52,53]. The EEG frequency and amplitude fall with age, especially in those with mild memory problems [54]. These problems may be partially solved by cholinesterase inhibitors that enhance alpha rhythms as flicker probably does [51,55]. There is a possibility that activity induced by flicker enhances mouse hippocampus activity and the human cortico-cortical and cortico-thalamic loops [56,57,58].

The use of the CFF was considered in subjects with obstructive sleep apnoea syndrome who may have cognitive impairment. This syndrome may affect attention, memory and executive functions that are associated with brain changes, especially in frontal regions [59]. Depending on the existence and size of brain lesions, CFF performance may vary, but the results of previous studies were inconclusive, and there is a need for future research with a larger sample size [60,61].

## 5. Diving and Hyperbaric Medicine

Neuropsychological assessment may be difficult in the underwater environment. The number of available tests for these conditions is limited so CFF seems to be a good solution due to its advantages. In recent years, many studies have focused on examining the impact of both recreational and professional diving on cognitive functions and take into consideration both acute and chronic effects.

CFF is widely used in experiments involving divers [34,62] and provides a reliable assessment that can be compared to psychometric testing (e.g., trail-making tasks or math processing) under normobaric—10 min of breathing air or oxygen in a quiet room at a constant temperature of 22 °C [35]—and hyperbaric-dry chamber—breathing air or enriched air nitrox for 20 min at 4 ATA—conditions [63]. A decrease in cerebral performance was reported in association with a decreasing level of CFF and vice versa [3,63]. Balestra and colleagues highlighted that breathing pure oxygen in normobaric conditions has an impact on CFF performance and proved that CFF results are dependent on cortical arousal because of increased brain blood flow in occipital regions and modification of pupil size induced by scopolamine, a muscarinic antagonist [64].

While breathing hyperbaric oxygen, it seems that neuronal excitability measured by CFF depends on the oxygen dose. During a study on professional military divers from the Special Forces, the CFF was increased while breathing a PPO_2_ of 2.8 ATA, which represents augmented neuronal excitability, while CFF was decreased at a PPO_2_ of 1.4 ATA, which represents attention and alertness deterioration [65]. Interestingly results at the lower PPO_2_ (1.4 ATA) in this group seem contradictory to those observed in recreational divers [35]. Differences in performance might be explained by investigated populations (elite, experienced, combat vs. occasional, recreational divers). Hyperbaric oxygen induces neuromuscular hyperexcitability in normal volunteers, while attenuates such an effect in elite military divers frequently exposed to oxygen and pressure [66].

Recreational diving (generally to 40 m of seawater, msw; in the mentioned literature the depth is 33 msw) may result in changes in the CFF performance even at 30 min post-dive [35,36,67].

Another risk in diving is nitrogen narcosis, which in the course of the hyperbaric exposure can be compared to alcohol intoxication and may cause a neurologic syndrome characterised by an impairment of cerebral performance or increased arousal [3]. The influence of the depth narcosis can be compared to the effect of a glass of Martini for every 15 m of depth [68]. Actually, the inert gas (nitrogen) penetration in the lipids of the brain’s nerve cells and interference with nerve cells’ signal transmission (according to the Meyer-Overton rule) might be further strengthened by the pressure effect [69]. Consequently, it is likely that the depth interval between subsequent “glasses of Martini” would be possibly presented with a smaller value and 10 m is probably more realistically correlated to the increase in the narcotic risk in diving. Impaired cerebral performance includes dysfunction of time perception, reaction speed and ability to think, calculate and react [70].

High-pressure nervous syndrome (HPNS) may appear especially in professional divers who dive deeper than 150 msw due to rapidly increasing pressure in the CNS during compression [20,71]. Nowadays, advanced diving equipment with closed-circuit breathing apparatuses allows recreational divers to also reach depths previously unreachable. Therefore, HPNS becomes a real hazard also for sport and recreational underwater activities [72].

The negative effects of HPNS (e.g., impairment of motor, sensory, behavioural and cognitive function) are known, but there is still a need to conduct future research to understand better its influence on humans. A significant decrease of CFF was observed in divers exposed to high pressures while breathing heliox at 62 ATA, which is equivalent to a depth of 610 m [73].

Quite recently, Ardestani and colleagues asked two groups of divers (reported or non-reported HPNS symptoms) to complete a few tests from the Physiopad package to measure their working memory, vigilance and decision making at 180 to 207 msw [20]. The Physiopad package includes HPNS questionnaires, a hand dynamometry test, a CFF test, an adaptive visual analog scale (AVAS), a simple math process (MathProc test), a perceptual vigilance task (PVT) and a time estimation task (time-wall). The CFF was performed daily and showed no differences between these two groups of divers, which means that the association between psychometric tests and subjective measurements may not exist. These results may arise from one of the limitations of this study; namely, none of the participants was medically diagnosed with HPNS.

Interestingly, the CFF correlates with hypoxemia, as shown during experimental exposures in hypobaric chambers for aviation purposes [74].

## 6. CFF and Its Connection with Brainwaves

CFF performance is related to other measures of brain activity. Some electrophysiological experiments have shown that the human visual cortex is sensitive to the frequency of flickering light, which induces neural activity changes in electroencephalogram (EEG) at the same level. Adrian and Matthews discovered “…that regular potential waves at frequencies other than 10 a second can be induced by flicker” [75] (p. 377). Authors recorded the EEG activity from the occipital lobe of subjects exposed to flickering light with frequencies up to 25 Hz, and that was the first time when steady-state visually evoked potentials were recorded [76,77].

In healthy older people, alpha-like EEG (8–12 Hz) can be induced by flicker and may even strengthen memory [78]. This relationship is highly specific for frequency. Williams et al. asked participants to identify the real word in 10 pairs of trigrams (three-letter words). After the practice task, the learning phase ensued (48 pairs of trigrams) [51]. Two minutes later the participants had to choose the “old” word in a pair of “old”-“new” trigrams (this time all of the words were real). Flickering light occurred in the learning phase (1000 ms in the beginning when the screen was blank); then the flicker was continued for 500 ms before and after the trigrams appeared. The frequency and intensity of flickers were randomised and unique for each participant. Six pairs of trigrams occurred after flickering at each frequency (two at each frequency-intensity combination). Results showed that flicker frequencies close to 10.0 Hz have a positive effect on recognition, unlike 8.7 and 11.7 Hz, which were ineffective.

Similar conclusions were made by Herrmann, who observed that 10 Hz visual stimulation has the strongest impact on the visual cortex and induces the strongest neural entrainment (brainwave frequency synchronisation) [79].

Sauseng et al. also showed the connection of alpha-frequency (around 10 Hz) stimulation with memory [80]. They concluded that repetitive transcranial magnetic stimulation might increase the capacity of short-term memory by enhancing the ability to ignore distractors. They emphasised that memory capacity also relied on a larger number of skills such as successful retention of the most important information as well as attention and executive functions.

## 7. Conclusions

To conclude, critical flicker fusion frequency has been a widely used method to assess cortical arousal and visual system parameters for many years. It may be successfully used in research involving both human and animal subjects. The CFF test may measure cognitive functioning just as well as other psychometric methods, although it must be considered carefully. As a simple and quick method, resistant to the learning effect, it may be used in many groups, from new-borns to the elderly, from healthy people to people with various diseases, such as epilepsy, dementia, minimal hepatic encephalopathy, etc. Despite these advantages, there is a need to conduct future research to compare the CFF test with other measures, especially neuropsychological ones, to verify its reliability and to better understand its advantages and limitations.

## Data Availability

No new data were created or analyzed in this study. Data sharing is not applicable to this article.
